# Cystic epithelioid malignant mesothelioma of the mesentery with rapid postoperative metastasis: an unusual case report

**DOI:** 10.3389/fmed.2026.1903020

**Published:** 2026-08-26

**Authors:** Ting Xu, Xue Meng, Xuan Gou, Xinyuan Wang, Shuai Luo, Deyuan Zhang

**Affiliations:** 1 Department of Pathology, Zhejiang Provincial People's Hospital Bijie Hospital (The First People’s Hospital of Bijie), Bijie City, Guizhou, China; 2 Department of Pathology, Affiliated Hospital of Zunyi Medical University, Zunyi City, Guizhou, China

**Keywords:** abdominal pain, asbestos, malignancy, malignant peritoneal mesothelioma, mesothelioma

## Abstract

**Background:**

Epithelioid malignant mesothelioma is a subtype of peritoneal mesothelioma associated with a relatively better prognosis. Herein, we report a case of epithelioid malignant mesothelioma in a 50-year-old Chinese female without a history of asbestos exposure. The tumor initially presented as a localized cystic mesenteric lesion. However, metastasis to the liver margin was detected 22 days after surgery, accompanied by rapid disease progression within one month. Gross examination revealed a cystic mass. Histologically, the tumor was consistent with epithelioid malignant mesothelioma, a rare pathological finding.

**Case demonstration:**

A 50-year-old female patient was admitted with a two-month history of right lower quadrant abdominal pain and no reported asbestos exposure. Ultrasound revealed a cystic structure of undetermined nature and origin in the right lower abdomen. Contrast-enhanced magnetic resonance imaging (MRI) of the mid-to-lower abdomen demonstrated a round mass adjacent to the cecum, measuring 47  ×  40  ×  59 mm. On T1-weighted imaging, the lesion exhibited high signal intensity on in-phase, out-of-phase, and fat-suppressed sequences; on T2-weighted imaging, it appeared isointense to slightly hyperintense. The mass wall was unevenly thickened with irregular margins. The central area of the lesion showed no enhancement and appeared hypointense. The mass was located in the pericecal region, suggesting a cystic mesenchymal tumor. Postoperative pathological examination confirmed the diagnosis of localized epithelioid malignant mesothelioma of the mesentery. Metastasis was detected 22 days after surgery.

**Conclusions:**

We report this rare case of localized malignant mesothelioma to raise awareness among clinical pathologists of this entity, facilitate accurate diagnosis, and help avoid misdiagnosis.

## Background

The serosa includes the pleura, peritoneum, and pericardium, which can give rise to benign tumors such as adenomatous tumors and malignant mesotheliomas (1). Malignant mesothelioma is a rare, aggressive tumor commonly found on the serosal surfaces, with an incidence of approximately one in a million (2). According to the literature, malignant peritoneal mesothelioma lacks specific symptoms, clinical features, and imaging characteristics. As a result, preoperative diagnosis is challenging and often leads to misdiagnosis (3). Cystic malignant peritoneal mesothelioma presenting as a localized mesenteric mass is even rarer, accounting for about 2% of the tumor's gross appearance (4).

## Case demonstration

A 50-year-old female patient was admitted due to right lower abdominal pain for 2 months. Physical examination on admission revealed right lower quadrant tenderness without rebound tenderness or muscle guarding. No palpable abdominal mass was detected.

Upon admission, an ultrasound examination revealed a cystic structure in the right lower abdomen measuring 51  ×  42 mm, with clear borders, a regular shape, and poor sound transmission within. The nature and origin of the mass remained unclear. MRI of the middle and lower abdomen (both plain and enhanced scans) showed a mass near the cecum, measuring 47  ×  40  ×  59 mm, presenting a round-like signal. On T1-weighted imaging, both the in-phase and out-of-phase images, as well as fat-suppressed images, showed high signals, while on T2-weighted imaging, the signal was isointense to slightly hyperintense. The lesion's anterior and superior areas showed enhancement with marginal necrosis, the wall thickness was uneven, and the lesion's central area showed no enhancement and appeared as low signal intensity. The mass was located near the cecum, suggesting a cystic mesenchymal tumor. The patient then underwent laparoscopic retroperitoneal tumor resection. Intraoperatively, the appendix appeared normal, while a mass was noted in the retroperitoneum near the cecum. The lateral peritoneum was opened, and the tumor was dissected along its margin. The tumor appeared cystic and was completely resected, presenting a gray-brown color. The tumor was sent for pathological examination.

Gross examination revealed a cystic mass attached to adipose tissue, measuring 60 × 47 × 40 mm. The mass had been previously opened clinically, and the cystic contents had leaked out. The inner wall of the cyst was smooth, with a wall thickness ranging from 2 to 28 mm, and areas of the wall were thickened, showing a gray-brown, solid texture.

**Figure 1:**
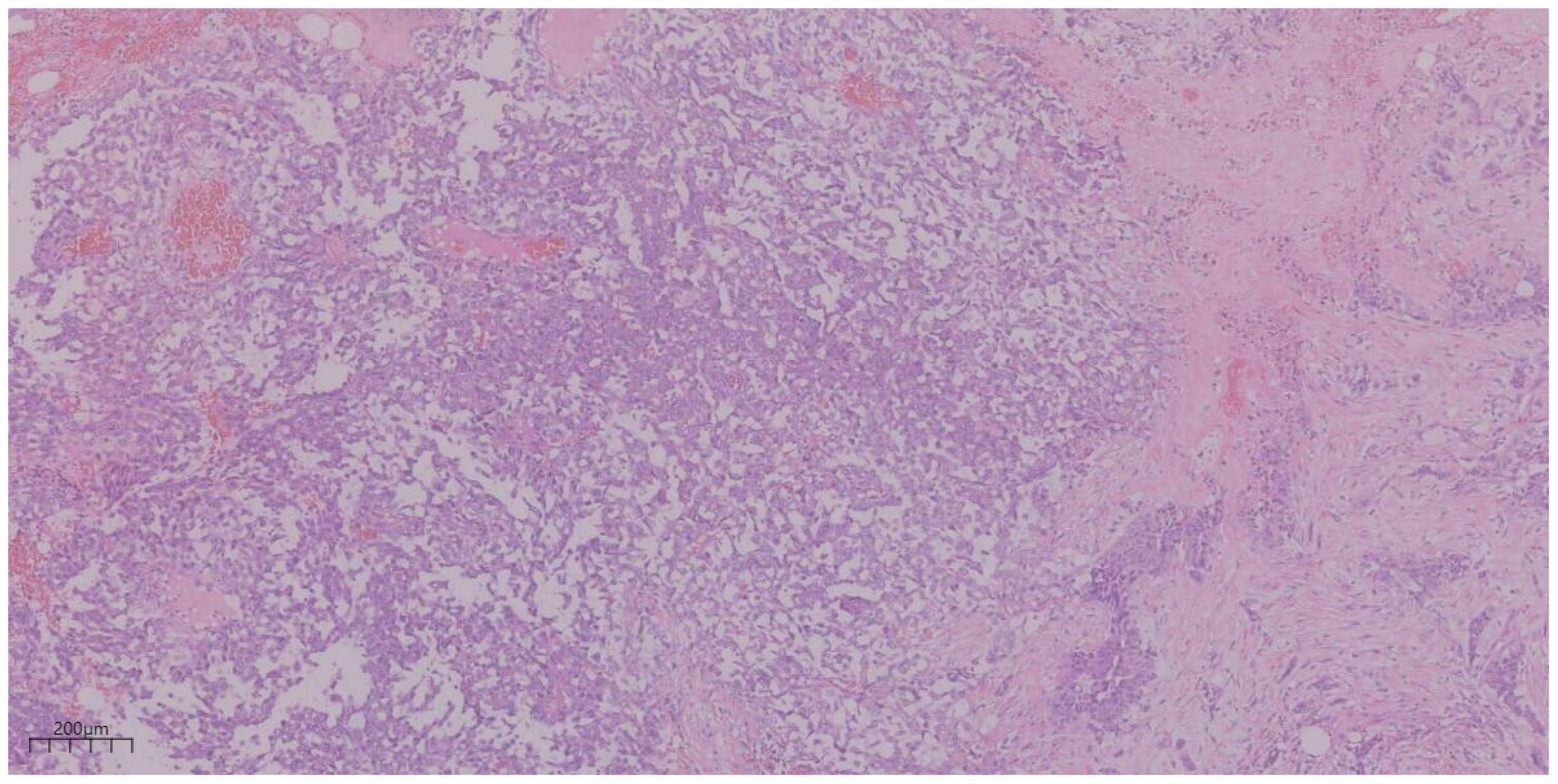
Under low-power microscopy, the tumor cells are composed of two different tumor cell structures. The tumor cells on the left are loose and have more bleeding. The tumor cells on the right side were in irregular cord-like, tubular and slit shapes, trapped in the tumor fibrous stroma, with no hemorrhage or necrosis observed (HE × 40).

**Figure 2:**
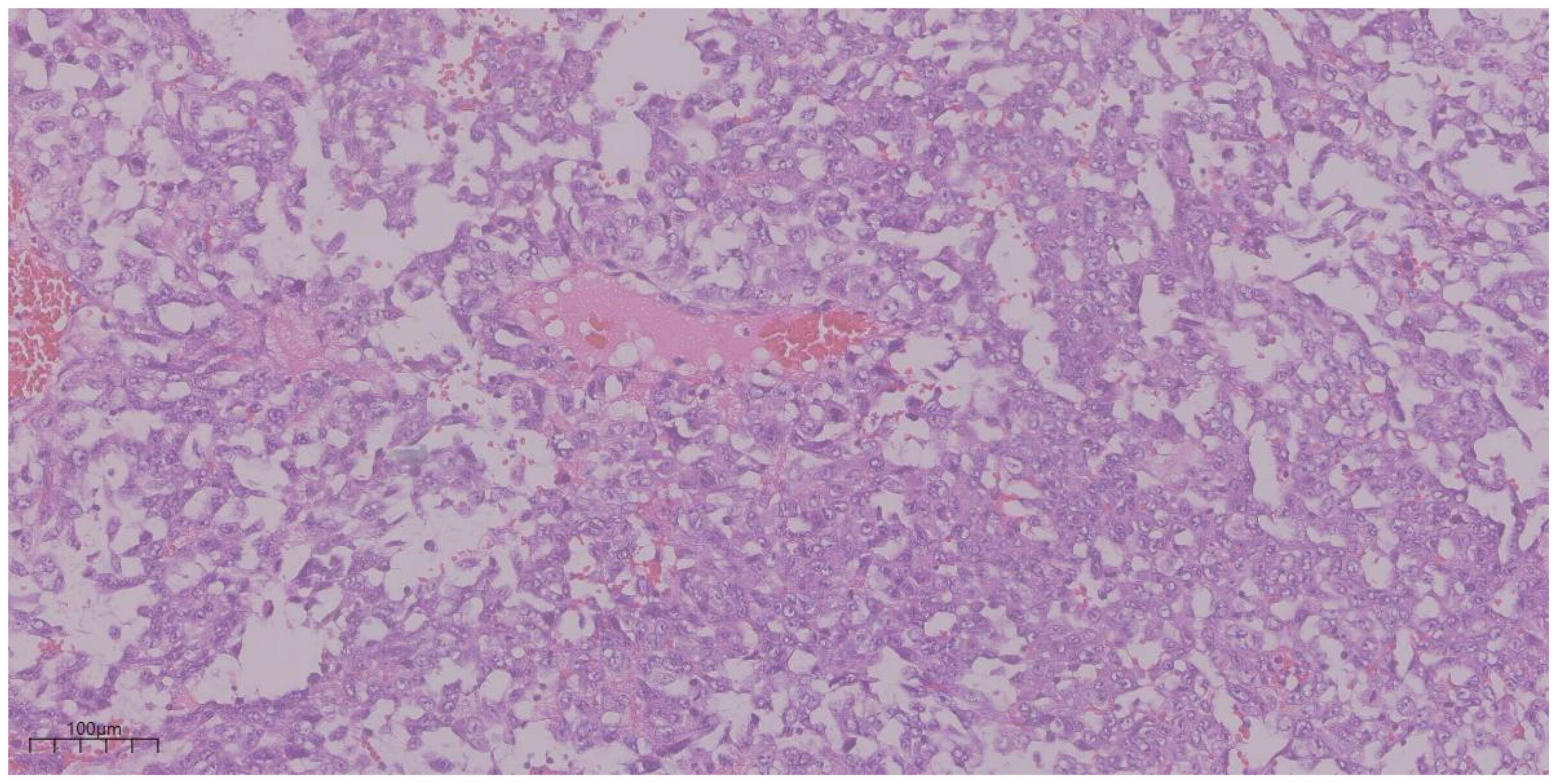
Under medium magnification, all tumor cells were epithelial-like, and a large number of vacuoles were seen (HE × 100).

Microscopically, at low magnification, the tumor cells were composed of two distinct cell structures. On the left side, the tumor cells were loosely arranged with considerable hemorrhage. On the right side, the tumor cells were arranged in irregular cords, tubules, and clefts, embedded in a fibrous tumor stroma, with no visible hemorrhage or necrosis (Figure 1). At medium magnification, the tumor cells were predominantly epitheliod, with numerous vacuoles observed on the left side (Figure 2). At high magnification, the tumor cells were round or oval, with slightly eosinophilic cytoplasm and prominent nucleoli, and mitotic figures were easily observed.

**Figure 3:**
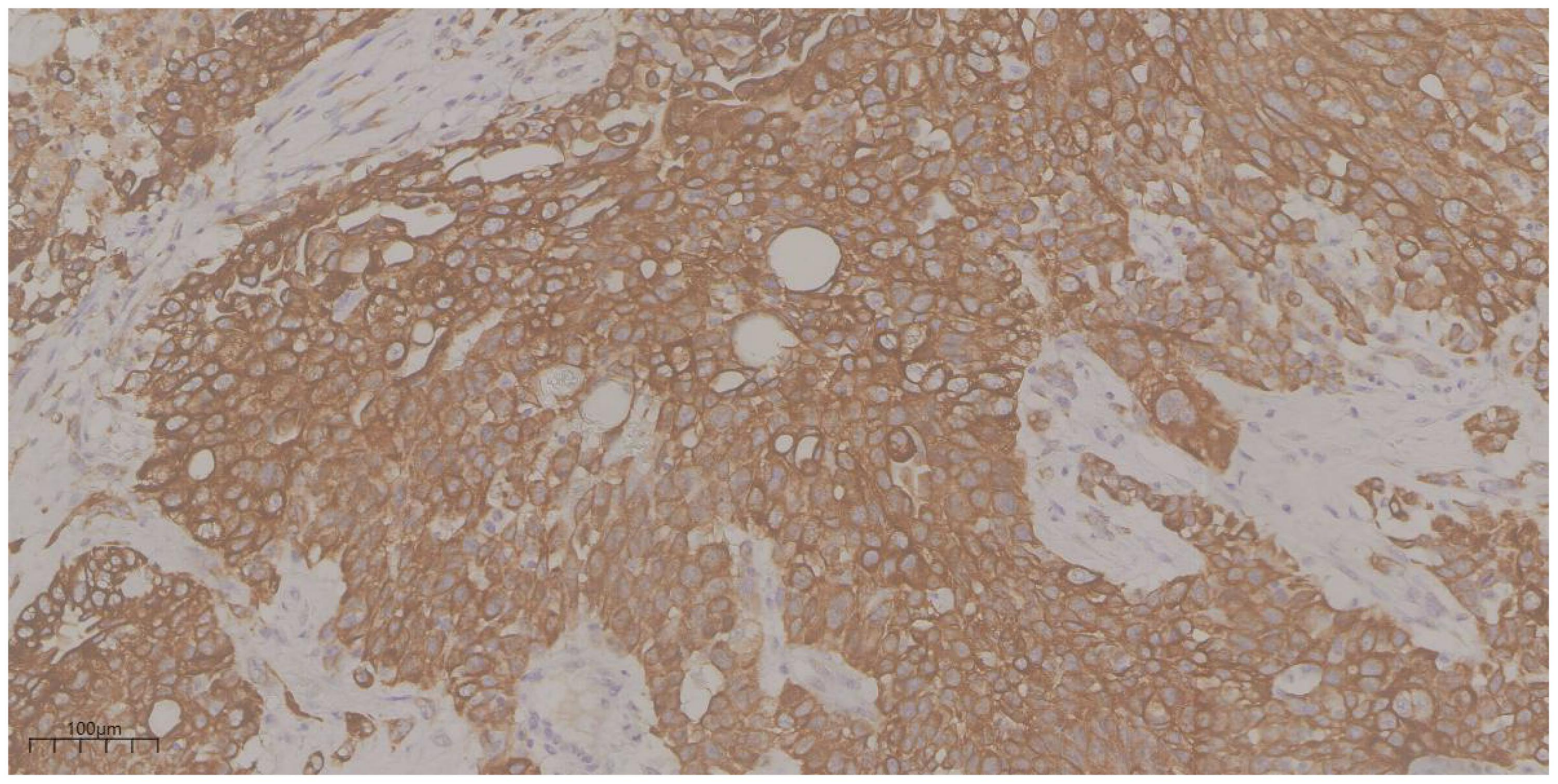
Immunohistochemistry showed positive expression of CKpan (×100).

**Figure 4:**
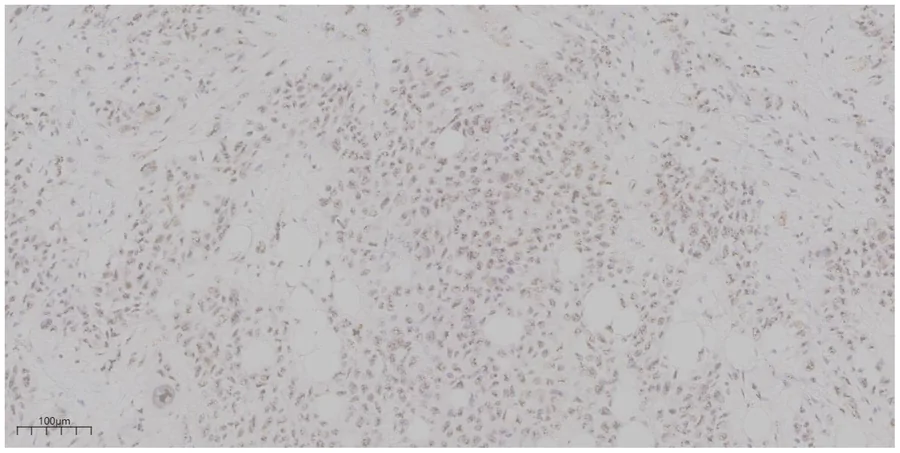
Positive expression of WT-1 by immunohistochemistry (×100).

**Figure 5:**
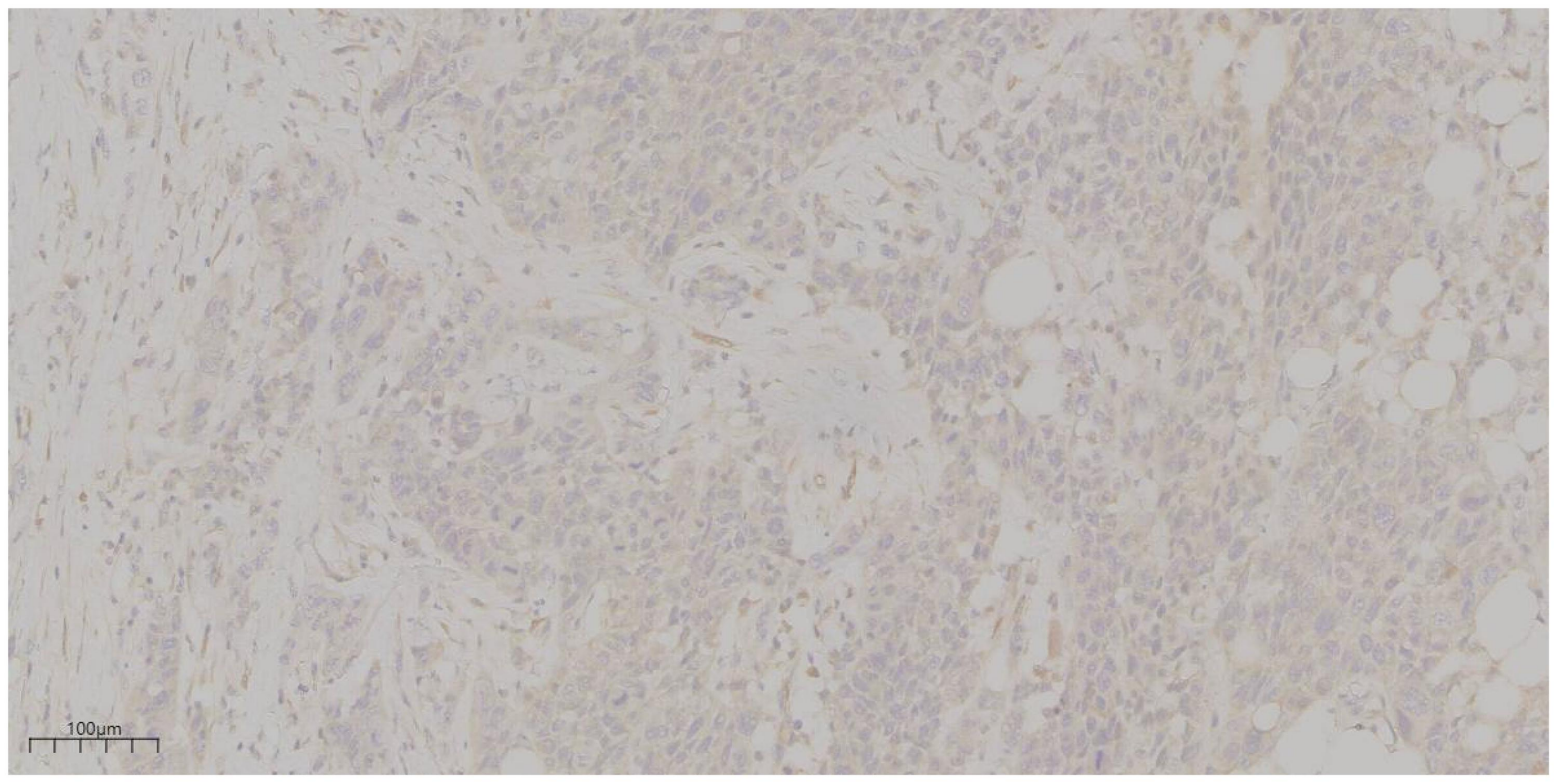
Immunohistochemical positive expression of CR (×100).

Immunohistochemical staining showed: CKpan (+) (Figure 3), WT-1 (+) (Figure 4), D2–40 (focally +), Calretinin (+) (Figure 5), CK5/6 (focally +), CK7 (−), ER (−), PR (−), P16 (−), MyoD1 (−), Myogenin (−), CD31 (−), CD34 (−), GATA3 (−), P40 (scattered +), CD99 (−), DOG-1 (−), CD117 (−), Ki-67 (60% in hotspot areas +).

Pathological diagnosis: Localized epitheliod malignant mesothelioma of the mesentery in the pericecal region.

Based on this case, the differential diagnosis should include the following: (1) Metastatic high-grade serous ovarian carcinoma: Given the patient's sex and the histological appearance under microscopy, ovarian origin should be considered. However, ultrasound showed no masses in either ovary, and immunohistochemistry revealed negative expression of P16 and PAX-8, which does not support metastasis from high-grade serous ovarian carcinoma. (2) Epitheliod mesenchymal tumor: Immunohistochemistry showed negative expression of CD117 and Dog-1, which ruled out an epitheliod mesenchymal tumor. Choriocarcinoma: Considering clinical testing for HCG, the patient's HCG level was 2.07 IU/L (non-pregnant range: <0.5–2.90 IU/L). Immunohistochemical staining for HCG should have been performed, but this test is unavailable in our department. (3) Epitheliod vascular tumors (e.g., hemangioendothelioma and angiosarcoma): These tumors express CD34, CD31, and ERG, which are usually absent in mesotheliomas.

22 days post-surgery, the patient presented with unexplained fever and underwent follow-up outpatient examination. CT imaging showed multiple patchy mixed-density lesions in the right mid-lower abdomen and under the liver margin, suggesting a space-occupying lesion with possible hemorrhage, raising concerns for tumor recurrence or metastasis. Following the CT findings, the patient was subsequently referred to the oncology department for further evaluation and management.

However, histopathological confirmation of these lesions was not obtained due to the patient's rapid clinical deterioration and institutional resource constraints at that time. Therefore, the diagnosis of metastasis in this case was based on radiological findings rather than pathological confirmation.

## Discussion

Local malignant mesothelioma, LMM) is a rare and under-recognized tumor (5). In the English literature, 101 cases of LMM have been reported. The tumor in these patients is localized and shares the same histopathological features as (diffuse malignant mesothelioma, DMM). The case we report not only reflects localized peritoneal malignant mesothelioma but also presents with cystic changes, which is even rarer in the literature. Some reports indicate that most malignant mesotheliomas arise from the malignant transformation of multicystic mesotheliomas (6). Local malignant mesothelioma (LMM) is a rare tumor, and its histopathological and immunophenotypic features are identical to those of DMM. In the two institutions involved in the IMP study, only 0.5%–1.6% of cases diagnosed as malignant mesothelioma were confirmed as LMM (7).

The age of patients ranges from 6 to 82 years, with 75% being male. Most (82%) of the tumors are located within the thoracic cavity (5). Other sites of involvement include the liver, mesentery, stomach, pancreas, umbilicus, spleen, and abdominal wall. Tumor size ranges from 0.6 to 15 centimeters. Most patients underwent surgical resection and/or chemotherapy or radiation therapy. In some patients, the median survival time was 29 months. Another 72 cases of LMM from the IMP institutions had an age range from 28 to 95 years, with 58.3% being male (5).

The patient in this case had no history of asbestos exposure. Among females, localized mesothelioma is more commonly seen in patients without a history of asbestos or other toxic substance exposure (8). This is consistent with reports in the literature.

Klemperer and Rabin further described five types of primary pleural tumors and classified them as localized or diffuse pleural tumors (9). The survival rate of localized malignant mesothelioma is significantly higher than that of diffuse malignant mesothelioma (10). The most common initial symptoms are abdominal pain (35%), abdominal swelling (31%), anorexia, significant weight loss, and ascites. Reports of night sweats and hypercoagulability are less common (11). Since abdominal pain is the most common presenting symptom, patients usually undergo laboratory tests and abdominal x-rays or CT scans (11). Serum mesothelin-related protein (SMRP) is a commonly elevated tumor marker, found in over 84% of mesotheliomas, with a sensitivity of 60% at diagnosis. CA-125, CA 15-3, hyaluronic acid, and osteopontin are other potential markers (12). Routine laboratory tests are not helpful in diagnosing mesothelioma. In this case, tumor marker tests were not performed. Currently, clinical examination mainly relies on imaging studies, but there is no specificity. CT findings may include peritoneal thickening, irregular and/or nodular masses, soft tissue masses in the abdominal or pelvic cavity, and/or thickening of the bowel wall (13).

This case did not undergo a CT scan, but MRI indicated a space-occupying lesion in the pericecal region. It is localized, without peritoneal thickening or nodular features. Currently, there are no effective tools for screening and early diagnosis of MPM, and most tumors are already in advanced stages when discovered. Additionally, it should be acknowledged that the suspected liver margin metastasis in this case was diagnosed based on CT imaging alone, without histopathological biopsy confirmation. While the radiological findings were highly suspicious for metastatic disease, pathological confirmation would have strengthened the diagnosis. Biopsy of suspected metastatic lesions should be pursued whenever clinically feasible.

Literature reports suggest that nuclear atypia and necrosis are independent prognostic factors in epitheliod mesothelioma, allowing it to be classified into low and high histological grades (14).

Due to the morphological patterns of MPM mimicking various epithelial and non-epithelial malignant tumors from a primary perspective, and considering the morphology of this case, differential diagnoses should include colorectal adenocarcinoma, mesenchymal tumors, angiosarcoma or epitheliod hemangioendothelioma, rhabdomyosarcoma, among others. Immunohistochemistry (IHC) is key in distinguishing these entities. In this case, considering factors such as gender, age, location, imaging findings, and histomorphology, immunohistochemical staining was performed to aid in diagnosis and differential diagnosis. However, no single immunohistochemical marker is sufficiently sensitive or specific for identifying MPM. Therefore, a panel of antibodies, including those for CK7, CD31, CD34, CD117, Dog-1, MyoD1, Myogenin, and mesothelial markers (WT1, calretinin, CK5/6, D2-40), was used for labeling. For the diagnosis of metastatic carcinoma, in addition to the aforementioned immunohistochemical tests, specific antibodies for the primary site, such as for ovarian serous carcinoma (ER, PR, PAX-8, P16), were added. In this case, the markers for ovarian serous carcinoma were negative. GATA-3 immunohistochemistry was also negative, ruling out breast cancer and urothelial carcinoma. GATA-3 may be positive in mesotheliomas, so it is recommended to combine the patient's history with relevant auxiliary examinations.

Although the immunohistochemical panel employed in this study successfully confirmed the diagnosis of epithelioid malignant mesothelioma and excluded the main differential diagnoses, it should be noted that BAP1 (BRCA1-associated protein 1) immunohistochemistry was not performed in this case. BAP1 is a tumor suppressor gene located at chromosome 3p21.1 that plays a pivotal role in maintaining genomic stability through its function as a deubiquitinating enzyme in DNA damage repair, cell cycle regulation, and chromatin remodeling (15). Loss of BAP1 protein expression is a common molecular event in malignant mesothelioma, with over 50% of peritoneal mesothelioma cases demonstrating loss of nuclear BAP1 expression. The reason BAP1 staining was not performed in this case is that this antibody is not yet included in our department's routine immunohistochemical panel, due to institutional resource constraints. We acknowledge this as a limitation of our study and suggest that BAP1 immunohistochemistry should be routinely considered in future cases of peritoneal mesothelioma to further confirm the diagnosis, assess prognosis, and guide potential targeted or immunotherapeutic approaches.

Furthermore, HCG immunohistochemistry was not performed in this case, as this antibody is not available in our department's routine immunohistochemical panel. Although choriocarcinoma was considered in the differential diagnosis and was reasonably excluded based on the patient's serum HCG level of 2.07 IU/L (within the non-pregnant reference range of <0.5–2.90 IU/L), we acknowledge that immunohistochemical confirmation would have provided greater diagnostic certainty. Ideally, HCG immunostaining should be included when choriocarcinoma enters the differential diagnosis, and its unavailability represents a technical limitation of our institutional resources.

Malignant mesothelioma typically lacks specific fusion genes in molecular genetics, with the most common molecular alteration being the inactivation or deletion of the BAP1 gene. Recent literature reports have shown that fusion genes such as *EWSR1::YY1* may be found in certain special cases (e.g., children or young patients) (16). Additionally, some studies have identified fusion genes such as *MAP3K8::ABLIM1* (17), *TNS3::MAP3K3*, and *ZFPM2::ELF5* (18) in multicystic mesotheliomas, suggesting that these may represent specific molecular subtypes. Fusion genes like *EWSR1::ATF1*, *FUS::ATF1*, and *EWSR1::NR4A3*, which are found in tumors with epithelioid morphology but atypical mesothelioma features, may have diagnostic value in differential diagnosis.these analyses were not applied to this case. The reason is that molecular testing (including next-generation sequencing and fusion gene detection) is not routinely available at our institution due to resource and equipment constraints. Such testing would be valuable in future cases to identify potential therapeutic targets, particularly in aggressive presentations like the one described here.

The treatment of MPM is based on a tri-modal approach: surgery, chemotherapy (neoadjuvant or adjuvant), and radiotherapy, particularly in patients without lymph node involvement (19). Studies have found that MPM has a better prognosis than sarcomatoid and mixed-type mesotheliomas. In this case, potential metastasis was found 22 days after surgery. Currently, assessing the treatment response of MPM remains challenging, especially due to the poor sensitivity and operator-dependence of radiologic evaluation (13).

Regarding the therapeutic strategy for this case, it should be noted that although cytoreductive surgery (CRS) combined with hyperthermic intraperitoneal chemotherapy (HIPEC) has been established as the first-line standard of care for diffuse malignant peritoneal mesothelioma (20), with studies demonstrating significant survival benefits in selected patients (1-year, 3-year, and 5-year survival rates of 70.3%, 65.6%, and 59.4%, respectively) (21) the present case was characterized by an exceptionally aggressive clinical course, with radiologically suspected liver margin metastasis detected as early as 22 days post-surgery. Even if CRS-HIPEC had been considered in the perioperative period, such rapid distant dissemination would have precluded timely implementation of this intervention. This observation suggests that for localized cystic mesothelioma with a high Ki-67 proliferation index (60%) and aggressive biological behavior, surgical resection alone may be insufficient to control micrometastatic disease, and early multidisciplinary team (MDT) involvement is essential for devising individualized comprehensive treatment strategies (22).

An additional factor that may have contributed to the unusually rapid postoperative progression in this case is the intraoperative or pre-operative rupture of the cystic mass. Macroscopic examination noted that the cyst had been “previously opened clinically” with extravasation of its contents. Rupture of a cystic mesothelioma could theoretically release viable tumor cells into the peritoneal cavity, facilitating peritoneal seeding and subsequent dissemination (23). This hypothesis is particularly relevant given that multicystic peritoneal mesotheliomas are known to have recurrence rates as high as 41%–50% (23, 24), and intraoperative spillage of cyst contents has been implicated as a risk factor for recurrence (25). While this remains speculative without direct pathological evidence of peritoneal seeding, it offers a plausible mechanism for the rapid metastatic course observed in this patient, and underscores the importance of meticulous surgical technique to avoid cyst rupture during resection.

## Conclusion

We report a case of localized malignant mesothelioma in the mesentery of the cecum, with no history of asbestos exposure, presenting clinically with abdominal pain as the primary symptom. Pathologically, the tumor appeared as a single mesenteric mass with cystic changes, which has not been previously reported in the literature. Histologically, it was diagnosed as epitheliod malignant mesothelioma with extensive necrosis and hemorrhage. The prognosis of this case is poor, with metastasis occurring in the short term. Therefore, necrosis and hemorrhage may be important factors in the prognosis assessment of MPM, although this needs to be confirmed by further studies in the literature.

## Data Availability

The original contributions presented in the study are included in the article/Supplementary Material, further inquiries can be directed to the corresponding author.
